# Differential Responses of the Ant *Odontoponera transversa* to Termite Chemical Signals: Evidence for Prey Preference

**DOI:** 10.3390/insects17050501

**Published:** 2026-05-14

**Authors:** Xiao-Lan Wen, Shu-Min Fan, Bao Jia, Yuan-Ru Wu, Zhao-Tian Li

**Affiliations:** 1 School of Preclinical Medicine, Guangxi Medical University, Nanning 530021, China; wenxiaolan@gxmu.edu.cn (X.-L.W.);; 2 Key Laboratory of Guangxi Colleges and Universities of Biological Molecular Medicine Research, Nanning 530021, China; 3 Nanning Institute of Termite Control, Nanning 530023, China; 4 Central Laboratory, School of Preclinical Medicine, Guangxi Medical University, Nanning 530021, China

**Keywords:** ant–termite interaction, chemical eavesdropping, prey preference, trail pheromone, *Odontoponera transversa*

## Abstract

Predators can exploit chemical signals of social insects to locate prey. Ants are considered as major predators of termites, and the interactions between them have been shaped by a long evolutionary history. Here, we investigated whether the ant *Odontoponera transversa* exhibits differential responses towards three termite species: *Coptotermes formosanus*, *Ancistrotermes dimorphus*, and *Macrotermes barneyi*. Through a combination of field trapping experiments and laboratory behavioral assays, we found that *O. transversa* showed stronger predatory responses towards *A. dimorphus* and *M. barneyi* than towards *C. formosanus*. Furthermore, when *A. dimorphus* or *M. barneyi* was present, attacks on *C. formosanus* were rarely observed, suggesting a hierarchy in prey preference. Behavioral assays indicated that termite trail pheromones serve as key cues influencing ant foraging decisions. Our findings suggest that differential responses to chemical signals, particularly trail pheromones, may underlie the observed prey preference patterns in *O. transversa*. Together, these results demonstrate that chemical signals, particularly trail pheromones, are key determinants of prey preference in *O. transversa*, offering a foundation for understanding the chemical ecology of ant–termite interactions.

## Simple Summary

Ants are important hunters of termites, but they do not attack all termite species equally. In this study, we asked why a common predatory ant, *Odontoponera transversa*, prefers some termites over others. We tested three termite species: two that are often found in forests and one, the Formosan subterranean termite (*Coptotermes formosanus*), which is a serious pest that damages wooden buildings and trees. We found that the ant readily attacked and ate the two forest termites but mostly ignored the pest termite. Even when the pest termite was placed together with a preferred one, the ant still chose the preferred species. We then discovered that the key difference lies in the chemical trails that termites use to communicate. The trail chemicals of the pest termite attracted very few ants, whereas those of the other two termites attracted many. Our results show that chemical signals determine the ant’s menu choice. Understanding this helps explain why the pest termite often escapes ant predation and may lead to new ways to protect buildings and forests using natural attractants or repellents.

## 1. Introduction

Predation is a fundamental driver of ecological and evolutionary dynamics, shaping community composition and population structure [1,2]. In predator–prey interactions, predators often exploit chemical signals of prey to enhance detection and localization. For instance, bats integrate multisensory cues to locate frogs more effectively than using single cues alone [3,4]. Such eavesdropping on communication signals is particularly relevant in social insects, which rely heavily on pheromones for colony coordination [5,6]. This reliance may inadvertently increase their vulnerability to predation, as predators can intercept these chemical cues [7]. Meanwhile, prey invest substantial energy in defense strategies—behavioral, mechanical, chemical, or combinations thereof—to avoid predation [7,8,9].

Ants and termites, both highly social insect groups with overlapping habitats and high biomass in tropical regions, share a long history of antagonistic interactions. Fossil evidence suggests that their predator–prey relationships date back approximately 11 million years [10]. Ants are widely considered as the most effective predators of termites, contributing significantly to termite mortality in many ecosystems [11,12]. Their overlapping foraging ranges bring them into frequent contact, intensifying these interactions [12,13].

Most termite species inhabit underground environments, have reduced vision, and rely on volatile or semi-volatile chemical signals—such as trail, alarm, and sex pheromones, as well as cuticular hydrocarbons—for intra-specific communication [14]. These chemical signals can be intercepted by predatory ants, providing reliable cues for prey localization. For example, the ant *Odontoponera transversa* (Hymenoptera: Formicidae: Ponerinae), a solitary hunter with robust bodies, strong fighting ability, and a powerful sting, has been shown to use termite trail pheromones as foraging cues [6,15]. Similar eavesdropping on termite alarm signals has also been documented in other ant species [16].

Although ants are important predators of termites, they exhibit marked differences in predatory preference towards different termite species [12,17,18]. In our preliminary observations, *O. transversa* readily attacked *Ancistrotermes dimorphus* and *Macrotermes barneyi* but rarely attacked *Coptotermes formosanus*.

What factors account for this differential predatory behavior? Here, we propose and test two non-mutually exclusive hypotheses to explain the differential responses of *O. transversa* to different termite species. Chemical signal hypothesis: Different termite species produce distinct chemical pheromones (e.g., trail or alarm pheromones) that serve as predatory cues, leading to differential attraction or repulsion by ants [19,20]. Behavioral defense hypothesis: Termite species differ in their defensive strategies and capabilities, which may influence ant predatory decisions as ants assess the risk or difficulty associated with subduing prey [21,22,23,24].

Understanding the mechanisms underlying the differential predation by *O. transversa*—particularly its unique response to *C. formosanus*—may provide insights into how predator perception of prey signals influences predator–prey dynamics and, more broadly, ecosystem stability [25].

## 2. Materials and Methods

### 2.1. Study Area

Field experiments and sample collections were conducted in eucalyptus and chestnut forests in Natong Town and Changtang Village, Nanning, Guangxi, as well as in the Liangfengjiang National Forest Park (LNFP) (22°43′28″ N, 108°16′59″ E; 70–220 m a.s.l.), Nanning. The region has a subtropical monsoon climate with an average annual temperature of 21.8 °C and average annual precipitation of 1286 mm. LNFP supports abundant subtropical biological resources and diverse ant communities, including populations of *Odontoponera transversa*.

### 2.2. Collection of Experimental Insects

*O. transversa* workers were collected using a specialized aspirator and transported to the laboratory in containers with moist cotton. The ant colony could be maintained stably in the laboratory for long-term rearing. Ants were maintained in artificial nests (20 cm × 12 cm) with separate brood, activity, foraging, excretion, and drinking areas. Approximately 15 workers from the same colony were housed per nest. A red transparent cover was placed over the nest to facilitate observation while minimizing disturbance.

Three termite species were used: *Coptotermes formosanus*, *Ancistrotermes dimorphus*, and *Macrotermes barneyi*. For each termite species, individuals were collected from at least three different colonies (nests) to ensure biological representativeness and to avoid colony-specific biases. For field trapping experiments using live termites, termite workers were collected on the morning of the trapping day. For laboratory behavioral experiments (e.g., predation behavior observation, choice assays), *A. dimorphus* and *M. barneyi* were collected from LNFP one day prior to the experiment and kept under laboratory conditions (25 ± 1 °C, moist filter paper) until use, to ensure viability and allow acclimation. *C. formosanus* was obtained from the Nanning Termite Control Research Institute, where it is maintained under laboratory conditions.

### 2.3. Field Trapping Experiments

**Figure 1:**
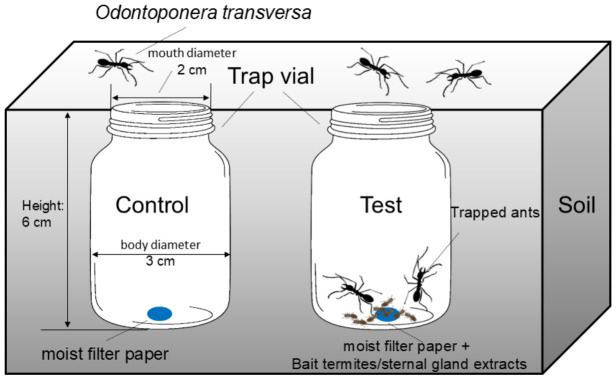
The device for trapping *O. transversa* in the field. The trap vial is directly buried in the ground, and the mouth of the vial is parallel to the surface of the ground. The trap vial can be freely chosen by the *O. transversa*. Once they fall into the trap vial, they cannot climb out.

The device for trapping (Figure 1) consisted of narrow-mouth cylindrical vials (mouth diameter: 2 cm, body diameter: 3 cm, height: 6 cm) with smooth inner walls to prevent ant escape. At each trapping site, two soil pits (diameter: 5 cm, depth: 10 cm) were dug 20 cm apart, and two vials were buried with their openings flush with the soil surface. Trapping was conducted on sunny days. Three trapping sites were located >1 km apart to ensure independence. Vials were deployed at 8:00 and the number of trapped ants was recorded at 18:00.

#### 2.3.1. Live-Termite Trapping

For each termite species (*C. formosanus*, *A. dimorphus*, and *M. barneyi*), one vial contained 15 live worker termites with moist filter paper, and the paired control vial contained only moist filter paper. The traps were set at the three sites as described above. The number of *O. transversa* workers trapped in each vial was recorded. The sample sizes were: *M. barneyi* and *C. formosanus*, n = 24 traps per species; *A. dimorphus*, n = 20 traps per species.

#### 2.3.2. Trail Pheromone Extract Trapping

Sternal gland extracts were prepared by dissecting the sternal glands from 12 worker termites of each species and extracting them in 1 mL of n-hexane for 24 h at 4 °C. Each trapping sample consisted of 12 sternal gland equivalents (i.e., 12 glands) applied to a piece of filter paper (1 cm × 1 cm). The solvent was allowed to evaporate for 5 min before the filter paper was placed into the vial. The control vial received the same volume of n-hexane alone on filter paper. Twenty independent traps per extract type were deployed as described above. The number of trapped ants was recorded after 10 h.

### 2.4. Predation Behavior Observation (Trajectory Experiment)

**Figure 2:**
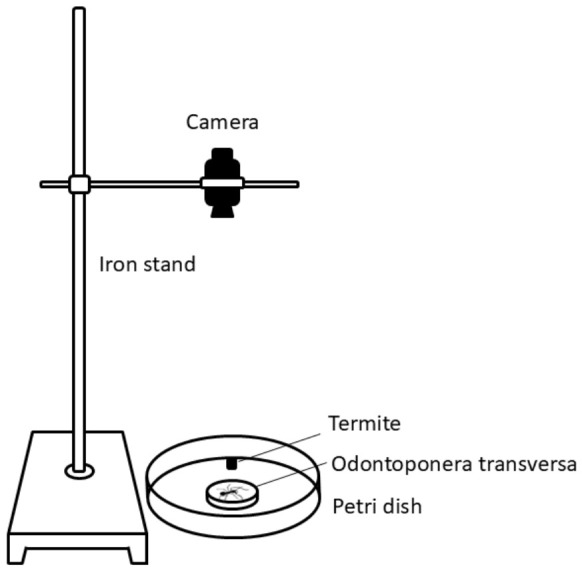
The setup of the trajectory experiment.

A camera was mounted on a stand facing downward over a 10 cm diameter Petri dish placed on a white background (30 cm × 30 cm). A 2 cm diameter Petri dish was inverted inside the larger dish as an acclimation chamber (Figure 2).

A single *O. transversa* worker was gently placed into the acclimation chamber using a brush. After the ant settled, the smaller dish was removed, and a single termite worker was introduced. Video recording (1 min duration) was started immediately. Sample sizes: *C. formosanus* (n = 24), *A. dimorphus* (n = 22), *M. barneyi* (n = 31), Trajectories were analyzed using Tracker video analysis and modeling software (version 6.x, Open-Source Physics) to extract movement speed and path patterns.

### 2.5. Predatory Behavior Experiments

#### 2.5.1. Predatory Responses to Single Termites

All ants and termites used in these experiments originated from at least three different field colonies (nests) to avoid colony-specific biases. Experiments were conducted in clean 10 cm diameter Petri dishes lined with moist filter paper (saturated with distilled water). A single *O. transversa* worker was introduced into the dish and allowed to acclimate for 2 min. Then, one termite worker of a given species (*C. formosanus*, *A. dimorphus*, or *M. barneyi*) was placed into the dish. The time from termite introduction to the first attack (defined as physical contact with an apparent attempt to kill) was recorded at 5, 10, 15, 20, and 30 min. This time-to-event data was used for survival analysis. In addition, the consumption status (whether the termite was partially or completely eaten) was checked at 8 h after the start of the trial. Ant survival was confirmed in all trials (100% survival). Termite status was recorded as alive, dead (from attack), or consumed (partial or full). No other time points were used for the consumption endpoint. Sample sizes: *C. formosanus* (n = 54), *A. dimorphus* (n = 54), *M. barneyi* (n = 60). All trials were conducted at room temperature (25 ± 1 °C) under ambient light.

#### 2.5.2. Prey Selection Between Two Termite Species

The experiments were conducted under the same conditions as described in Section 2.5.1 (clean 10 cm Petri dishes lined with moist filter paper saturated with distilled water). A single *O. transversa* worker was introduced and allowed to acclimate for 2 min. For each pairwise combination (*C. formosanus* vs. *A. dimorphus* and *C. formosanus* vs. *M. barneyi*), one worker of each termite species was simultaneously placed into the Petri dish. The first species attacked (initial predatory attempt) was recorded within the first minute after termite introduction. In addition, the consumption status (whether each termite was partially or completely eaten) was checked at 8 h after the start of the trial. Trials where no attack occurred within the first minute were recorded as “no attack” (both species unattacked). Trials where both species were attacked or consumed were also recorded accordingly. Each pairwise combination was replicated 30 times (n = 30 per combination). All trials were conducted at room temperature (25 ± 1 °C) under ambient light.

### 2.6. Biological Comparisons Among Termite Species

#### 2.6.1. Trail Pheromone Trapping

To test the chemical signal hypothesis, trail pheromone extracts were prepared and tested following the same protocol as described in Section 2.3.2. Briefly, sternal glands were dissected from 12 worker termites of each species (*C. formosanus*, *A. dimorphus*, and *M. barneyi*) and extracted in 1 mL of n-hexane for 24 h at 4 °C. Each trap received a filter paper (1 cm × 1 cm) impregnated with 12 sternal gland equivalents. The solvent was allowed to evaporate for 5 min before the paper was placed into the vial. A control vial containing the same volume of pure n-hexane on filter paper was used. Twenty independent traps were deployed per extract type, and the number of trapped *O. transversa* workers was recorded after 10 h, following the same field trapping arrangement as in Section 2.3.

#### 2.6.2. Defensive Capabilities of Soldier Termites

To test the behavioral defense hypothesis, experiments were conducted in 10 cm Petri dishes lined with moist filter paper (saturated with distilled water). A single *O. transversa* worker was introduced and allowed to acclimate for 2 min. Then, 1, 3, or 5 soldier termites of a given species (*A. dimorphus*, *M. barneyi*, or *C. formosanus*) were placed into the dish (soldier-to-ant ratios 1:1, 3:1, and 5:1). All soldier termites originated from at least three different field colonies per species to ensure representativeness. The survival status (alive or dead) of soldier termites and the ant was recorded after 8 h. Soldiers were considered alive if they showed any visible movement; dead if motionless and unresponsive to gentle probing. The number of replicates per species for each ratio (for 1:1 and 1:3 ratios, 18 replicates each; for 1:5 ratio, 12 replicates each). Under all tested conditions, the ant survived in every replicate.

### 2.7. Statistical Analysis

All statistical analyses were performed using GraphPad Prism 9.5 (GraphPad Software, San Diego, CA, USA) and SPSS 26.0 (for Fisher’s exact tests), with a two-tailed significance level set at α = 0.05. Non-parametric tests were used for count data or when normality assumptions were violated. (i) For field trapping experiments, Mann–Whitney U tests compared the number of ants trapped between treatment and control vials for each termite species. To compare trapping efficacy among the three termite species in both live-termite and pheromone-extract assays, Kruskal–Wallis tests were followed by Dunn’s multiple pairwise comparisons with Bonferroni correction. The same non-parametric procedure was applied to ant movement speed data. (ii) Kaplan–Meier survival curves were estimated for termite mortality within 30 min, and differences among species were tested using the log-rank (Mantel–Cox) test, with pairwise comparisons adjusted by Bonferroni correction. Individuals alive at the end of the trial were treated as censored. (iii) Consumption rates at 8 h were compared using a 3 × 2 Fisher’s exact test, followed by pairwise Fisher’s tests with Bonferroni correction. For dual-choice experiments, only discordant trials (where only one species was attacked or consumed) were analyzed. Attack preference was tested with exact binomial tests (null: 0.5). Consumption data involving double-positive outcomes were analyzed with McNemar’s test (R 4.2.0). (iv) Soldier survival at each ant-to-soldier ratio (1:1, 1:3, 1:5) was analyzed using separate 3 × 2 Fisher’s exact tests (SPSS 26.0); no correction was applied across ratios because they represent independent conditions. (v) Ant movement trajectories were qualitatively described using Tracker software (v6.1.1, Open-Source Physics).

## 3. Results

### 3.1. Field Trapping of O. transversa by Different Termite Species

**Figure 3:**
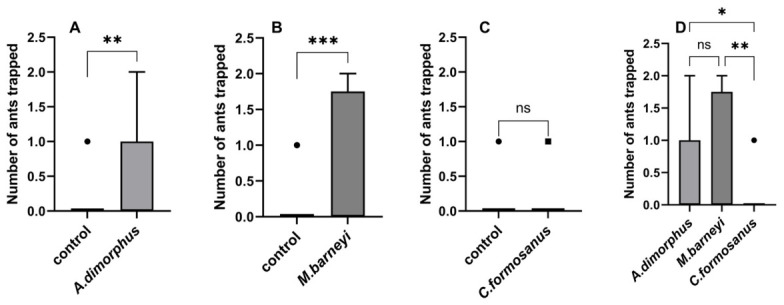
Field trapping of *Odontoponera transversa* by three termite species. (**A**–**C**) Box plots showing the number of ants trapped in treatment vials (containing termites) versus control vials (empty) for (**A**) *Ancistrotermes dimorphus*, (**B**) *Macrotermes barneyi*, and (**C**) *Coptotermes formosanus*. Horizontal lines indicate medians; points represent individual trap replicates. Mann–Whitney U tests: ** *p* < 0.01, *** *p* < 0.001, ns = not significant. Sample sizes: *A. dimorphus*, n = 20 per group; *M. barneyi* and *C. formosanus*, n = 24 per group. (**D**) Comparison of ant trapping numbers among the treatment groups. Boxes represent interquartile range (IQR), horizontal lines medians, points all replicates. A Kruskal–Wallis test followed by Dunn’s post hoc test with Bonferroni correction was used; * *p* < 0.05, ** *p* < 0.01, ns = not significant.

For each termite species, the number of ants trapped in treatment vials (containing termites) was compared with that in empty control vials. The treatment groups of *M. barneyi* (U = 165, n = 24 per group, *p* = 0.0009) and *A. dimorphus* (U = 119, n = 20 per group, *p* = 0.0068) trapped significantly more ants than their respective controls, whereas no significant difference was found for *C. formosanus* (U = 288, n = 24 per group, *p* > 0.9999; Figure 3A–C). To directly compare the attractiveness among the three termite species, we analyzed only the treatment group data. A Kruskal–Wallis test revealed a significant difference among species (H = 12.41, df = 2, *p* = 0.0020; Figure 3D). Post hoc comparisons with Bonferroni correction showed that *C. formosanus* attracted significantly fewer ants than both *A. dimorphus* (adjusted *p* = 0.0189) and *M. barneyi* (adjusted *p* = 0.0034), while there was no significant difference between *A. dimorphus* and *M. barneyi* (adjusted *p* > 0.9999) (Figure 3).

### 3.2. Ant Movement Speed and Trajectories upon Encountering Termites

**Figure 4:**
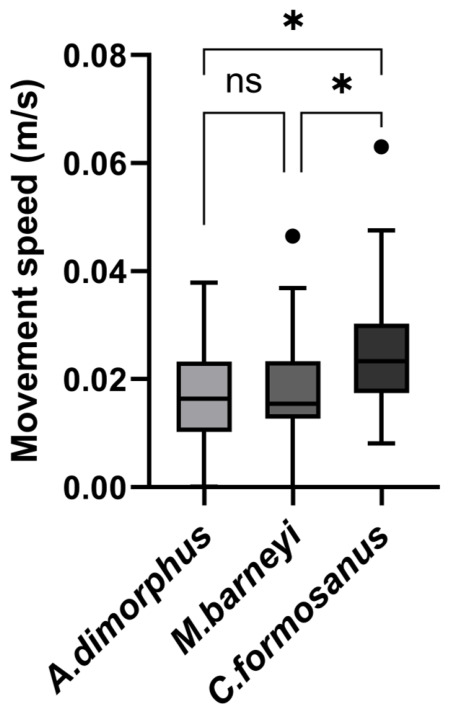
Movement speed of *Odontoponera transversa* upon encountering three termite species. Boxes represent the interquartile range (IQR), horizontal lines indicate medians, and points show individual replicates. A Kruskal–Wallis test followed by Dunn’s post hoc test with Bonferroni correction was used. * *p* < 0.05, ns = not significant. Sample sizes: *A. dimorphus* n = 24, *M. barneyi* n = 31, *C. formosanus* n = 24.

The movement speed of *O. transversa* differed significantly among the three termite species (Kruskal–Wallis test: H = 8.923, df = 2, *p* = 0.0115; Figure 4). Dunn’s post hoc comparisons with Bonferroni correction showed that ants moved significantly faster when encountering *C. formosanus* than when encountering *A. dimorphus* (adjusted *p* = 0.0287) or *M. barneyi* (adjusted *p* = 0.0252). No significant difference was detected between *A. dimorphus* and *M. barneyi* (adjusted *p* > 0.9999) (Figure 4).

**Figure 5:**
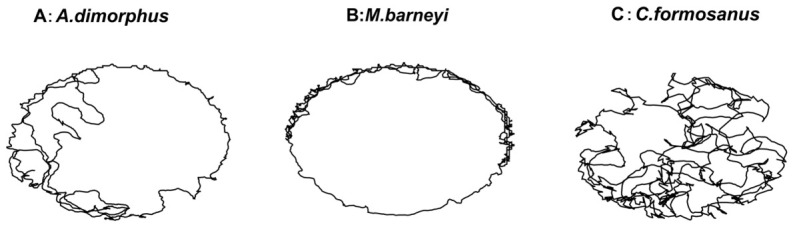
Representative movement trajectories of *Odontoponera transversa* when encountering different termite species. (**A**) *Ancistrotermes dimorphus*; (**B**) *Macrotermes barneyi*; (**C**) *Coptotermes formosanus*. Each trajectory was recorded over 1 min after the ant encountered a single termite worker. Ants moved slowly and remained near *A. dimorphus* and *M. barneyi*, whereas they moved rapidly and erratically around *C. formosanus*. Scale bars indicate 1 cm.

Trajectory analysis (Figure 5) qualitatively supported the speed results: when encountering *A. dimorphus* (Figure 5A) or *M. barneyi* (Figure 5B), ants walked slowly and remained near the termite, consistent with an active hunting posture. In contrast, upon encountering *C. formosanus* (Figure 5C), ants moved rapidly with irregular, winding paths, rarely approaching the termite.

### 3.3. Differential Predatory Responses of O. transversa to Three Termite Species

#### 3.3.1. Attack and Feeding on Single Termites

**Figure 6:**
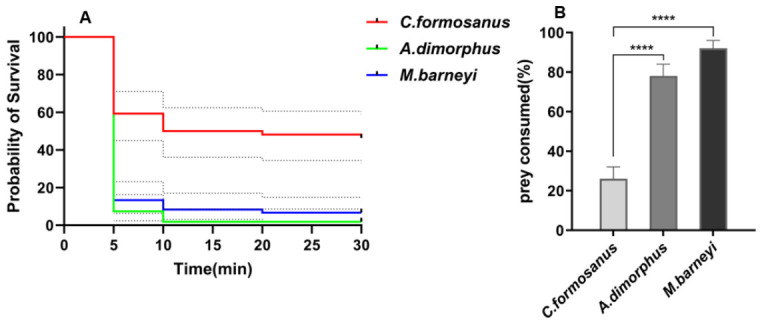
Attack-induced death dynamics and 8 h consumption of three termite species by *Odontoponera transversa*. (**A**) Kaplan–Meier survival curves showing the time to attack-induced death. Tick marks indicate censored individuals (alive at 30 min). Statistical comparisons: log-rank test with Bonferroni correction. Grey dotted lines represent the 95% confidence intervals for the survival curves. (**B**) Proportion of termites consumed within 8 h, expressed as a percentage. “Consumed” refers to termites that were partially or completely eaten by ants, regardless of the time of death. Bars represent mean ± SEM. Fisher’s exact test with Bonferroni correction was used; **** *p* < 0.001. Sample sizes: *C. formosanus* n = 54, *A. dimorphus* n = 54, *M. barneyi* n = 60.

The survival analysis showed a significant difference in the time until attack induced death among the three termite species (log rank test: χ^2^ = 50.59, df = 2, *p* < 0.0001; Figure 6A). *Odontoponera transversa* had a median survival time of 15 min, whereas both *Ancistrotermes dimorphus* and *Macrotermes barneyi* had a median survival of 5 min. Pairwise comparisons with Bonferroni correction confirmed that *C. formosanus* survived significantly longer than *A. dimorphus* and *M. barneyi* (adjusted *p* < 0.001 for both). No significant difference was found between *A. dimorphus* and *M. barneyi* (adjusted *p* > 0.05). The numbers of censored individuals (alive at 30 min) were 25 for *C. formosanus*, 1 for *A. dimorphus*, and 4 for *M. barneyi*.

The 8 h consumption rate also differed significantly among the three species (Fisher’s exact test, *p* < 0.0001; Figure 6B). *C. formosanus* was consumed in only 25.9% of trials (14/54), which was significantly less than *A. dimorphus* (77.8%, 42/54; adjusted *p* < 0.001) and *M. barneyi* (91.7%, 55/60; adjusted *p* < 0.001). No significant difference was detected between *A. dimorphus* and *M. barneyi* after Bonferroni correction (adjusted *p* = 0.165). These results indicate that *C. formosanus* is both attacked more slowly and consumed less frequently than the other two termite species. (Figure 6A,B; Table 1).

#### 3.3.2. Prey Selection Between Two Termite Species

**Figure 7:**
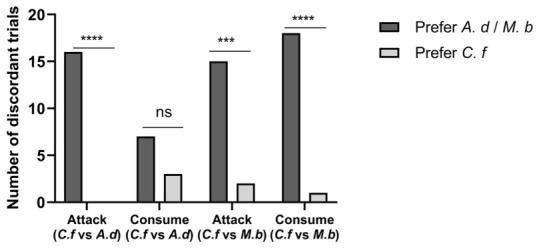
Prey selection by *Odontoponera transversa* in two-choice assays (n = 30 trials per pairwise combination). Bars represent the number of discordant trials—trials where only one termite species was attacked (first predatory attempt, left four groups) or consumed within 8 h (right four groups). Trials where both or neither species were attacked/consumed are excluded from analysis. Statistical comparisons: exact binomial test (attack) and McNemar’s test (consumption), both with Bonferroni correction. **** *p* < 0.0001, *** *p* < 0.001, ns = not significant. Error bars not applicable because each bar represents a single aggregated count.

In the two choice assays, the first predatory attempt (first attack) and 8 h consumption was recorded (Figure 7). When *C. formosanus* was paired with *A. dimorphus*, ants attacked *A. dimorphus* alone in 16 trials and never attacked *C. formosanus* alone (0 trials). This preference was highly significant (binomial test, *p* = 0.00018). For consumption, discordant trials comprised 7 trials where only *A. dimorphus* was consumed and 3 trials where only *C. formosanus* was consumed; this difference was not statistically significant (McNemar χ^2^ = 0.9, df = 1, *p* = 0.3428).

When *C. formosanus* was paired with *M. barneyi*, ants attacked *M. barneyi* alone in 15 trials and *C. formosanus* alone in 2 trials (binomial test, *p* = 0.0015). For consumption, discordant trials were 18 for *M. barneyi* alone and 1 for *C. formosanus* alone (McNemar χ^2^ = 13.47, df = 1, *p* = 0.00024). Together, these results show that *C. formosanus* was significantly less likely to be chosen as the first predatory target compared to both *A. dimorphus* and *M. barneyi*. However, its consumption disadvantage was significant only when paired with *M. barneyi*, not with *A. dimorphus*.

### 3.4. Trail Pheromones and Defensive Capabilities

#### 3.4.1. Trail Pheromone Trapping

**Figure 8:**
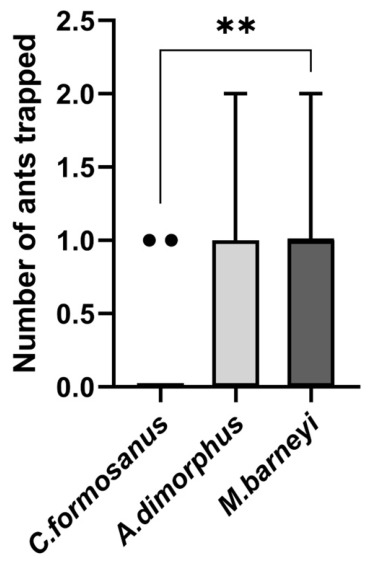
Number of *Odontoponera transversa* trapped using trail pheromone extracts from three termite species. Extracts were prepared from sternal glands of 12 workers per species. Each point represents an independent trap replicate (n = 20 per extract type). Boxes represent the interquartile range (IQR), horizontal lines the median, whiskers the Tukey range, and points all individual values. A Kruskal–Wallis test followed by Dunn’s post hoc test with Bonferroni correction was used. ** *p* < 0.01.

The sternal gland extract of *M. barneyi* attracted significantly more *O. transversa* workers than that of *C. formosanus* (Dunn’s test with Bonferroni correction, adjusted *p* = 0.0067; Figure 8). The overall difference among the three species was significant (Kruskal–Wallis test: H = 9.36, df = 2, *p* = 0.0093). No significant differences were detected between *A. dimorphus* and *M. barneyi* (adjusted *p* = 0.3102) or between *A. dimorphus* and *C. formosanus* (adjusted *p* = 0.4589). These results indicate that the trail pheromone of *C. formosanus* is less attractive to *O. transversa*, while *A. dimorphus* did not differ from *C. formosanus* in this isolated cue assay (Figure 8).

#### 3.4.2. Defensive Capabilities of Soldier Termites

When a single *O. transversa* was confronted with 1, 3, or 5 soldier termites of each species, no significant differences in soldier survival were detected among the three termite species (Fisher’s exact test: 1:1, *p* = 0.774; 1:3, *p* = 0.856; 1:5, *p* = 0.856; Table 2). Under all tested conditions, the ant survived in every replicate. These results indicate that soldier-based defense does not contribute to the observed differences in prey preference.

## 4. Discussion

In this study, we combined field trapping experiments and laboratory behavioral assays to investigate the differential predatory responses of *Odontoponera transversa* towards three termite species: *Coptotermes formosanus*, *Ancistrotermes dimorphus*, and *Macrotermes barneyi*. Our results consistently show that *O. transversa* exhibits significantly weaker predatory responses towards *C. formosanus* compared to *A. dimorphus* and *M. barneyi*. Field trapping revealed that *C. formosanus* attracted fewer ants than the other two species. Behavioral tracking showed that when encountering *C. formosanus*, ants moved faster and with more erratic trajectories, in contrast to the slow, oriented movements observed toward *A. dimorphus* and *M. barneyi*. Attack and feeding experiments further confirmed that *C. formosanus* was less frequently attacked and consumed. Moreover, in pairwise choice tests, ants consistently preferred *A. dimorphus* or *M. barneyi* over *C. formosanus*. Taken together, these findings demonstrate that *O. transversa* exhibits a clear prey preference hierarchy, with *C. formosanus* being the least preferred prey among the three species tested.

### 4.1. Chemical Signals Mediate Differential Prey Preference

Our results support the chemical signal hypothesis, which posits that differential ant responses to termite species are mediated by species-specific chemical cues. Termites rely heavily on volatile and semi-volatile pheromones for communication, including trail, alarm, and sex pheromones [14]. These chemical signals can be intercepted by predatory ants and used as foraging cues [6]. In our trail pheromone trapping experiment, sternal gland extracts of *C. formosanus* attracted significantly fewer *O. transversa* than those of *M. barneyi*, mirroring the results of live-termite trapping experiments. This suggests that chemical cues alone are sufficient to explain the observed differences in ant attraction.

The chemical composition of trail pheromones differs among termite species. The trail pheromone of *C. formosanus* consists of (Z,E,E)-dodeca-3,6,8-trien-1-ol and (Z,Z,E)-dodeca-3,6,8-trien-1-ol, whereas *A. dimorphus* uses (Z,Z)-dodeca-3,6-dien-1-ol, and *M. barneyi* uses (Z)-dodec-3-en-1-ol [15,17,18]. These structural differences—namely, the number and position of double bonds, as well as the degree of unsaturation—can significantly alter the molecular shape, polarity, and volatility of the pheromones. Such variations are known to affect the binding affinity of these compounds to odorant receptors (ORs) in ant antennae, thereby influencing olfactory detection and subsequent behavioral responses. Specifically, the more unsaturated and complex structure of *C. formosanus* pheromones may reduce their compatibility with the ORs of *O. transversa*, leading to weaker or no activation. These structural differences may underlie the differential attractiveness of these pheromones to *O. transversa*. The trail pheromone of *C. formosanus* may be less attractive to *O. transversa* due to reduced signal strength or different molecular structure. Alternatively, it could contain compounds that actively repel ants, although our data do not distinguish between reduced attraction and active repellence. Future studies using heterologous expression of ant odorant receptors or electroantennography (EAG) with synthetic compounds are required to directly test how each pheromone component is perceived at the receptor level.

### 4.2. Behavioral Defense Is Unlikely to Explain Prey Preference

We also tested the behavioral defense hypothesis, which proposes that termite defensive capabilities influence ant predatory decisions. When *O. transversa* was confronted with soldier termites at soldier-to-ant ratios of 1:1, 3:1, and 5:1, we observed no significant differences in soldier survival among the three species. All ants survived across all treatments, indicating that soldier defense did not pose a substantial threat to *O. transversa* under our experimental conditions. These results suggest that differential predation is unlikely to be driven by soldier-mediated defensive capabilities. Other defensive strategies, such as behavioral responses of workers (e.g., rapid retreat or aggressive biting), were not systematically quantified in this study and warrant further investigation.

### 4.3. Ecological Implications of Reduced Predation on C. formosanus

*C. formosanus* is one of the most destructive termite species, causing substantial damage to urban, agricultural, and forestry ecosystems in tropical and subtropical regions [26]. Its success as an invasive pest has been attributed in part to its ability to establish large colonies with few effective natural enemies. Our findings suggest that *O. transversa*, a common predatory ant in the region, does not pose a strong predatory threat to *C. formosanus*. This reduced predation pressure may contribute to the ecological success of *C. formosanus* in areas where *O. transversa* is abundant. Conversely, *A. dimorphus* and *M. barneyi*, which were readily preyed upon, may experience stronger top-down regulation by ants. Understanding the chemical basis of differential predation could inform biocontrol strategies, for instance, by identifying attractant pheromones that could be used to enhance ant predation on pest termites.

### 4.4. Limitations and Future Directions

Several considerations should be noted when interpreting our findings. First, while our laboratory experiments were conducted under simplified conditions, the consistency between field trapping and laboratory results suggests that the observed patterns are robust. Second, ants were maintained without brood, which may influence hunting motivation, yet the clear differences among termite species remain evident under these conditions. Third, while our pheromone trapping experiment supports the chemical signal hypothesis, future studies combining electrophysiological assays and two-choice olfactometer experiments could further establish causal links between specific pheromone components and ant behavioral responses. Testing synthetic versions of the three termite trail pheromones in such assays would directly reveal whether the observed differential attraction is due to receptor-level discrimination. Addressing these aspects will provide a more complete understanding of the chemical ecology underlying ant–termite interactions. Ant–termite interactions are not limited to predation; cohabitation and nesting associations have also been documented [27].

### 4.5. Conclusions

In conclusion, this study demonstrates that *O. transversa* exhibits differential predatory responses toward three termite species, with *C. formosanus* being significantly less preferred than *A. dimorphus* and *M. barneyi*. Our results support the chemical signal hypothesis: species-specific trail pheromones appear to mediate ant attraction and prey preference, whereas soldier-based defensive capabilities do not explain the observed patterns. These findings highlight the importance of chemical eavesdropping in shaping predator–prey interactions between ants and termites and suggest that chemical signaling plays a key role in determining prey susceptibility to predation. Understanding these mechanisms not only advances our knowledge of ant–termite ecology but also may inform the development of pheromone-based approaches for termite management.

## Figures and Tables

**Table 1:** Attack and killed (5 min) and feeding (8 h) on three species of termites by the *O. transversa*.

	n	Death (5 min)	Fed upon (8 h)
*C. formosanus*	54	22	14
*A. dimorphus*	54	50	42
*M. barneyi*	60	52	55

**Table 2:** Defensive capabilities of soldier termites against *O. transversa*.

Ant: Soldier Ratio	Termite Species	Soldiers Surviving/Total Replicates	*p* (Fisher’s Exact)
1:1	*A. dimorphus*	1/18	0.774
	*M. barneyi*	1/18	
	*C. formosanus*	2/18	
1:3	*A. dimorphus*	2/18	0.856
	*M. barneyi*	2/18	
	*C. formosanus*	3/18	
1:5	*A. dimorphus*	3/18	0.856
	*M. barneyi*	2/12	
	*C. formosanus*	2/12	

For each ant to soldier ratio, the number of soldier termites surviving after 8 h of co-exposure with a single ant worker is shown as “surviving/total replicates”. Fisher’s exact test was used to compare survival among the three termite species for each ratio independently. *p* values are reported exactly as calculated; no adjustment for multiple comparisons was applied because the ratios represent different experimental conditions. Ants survived in all replicates.

## Data Availability

The original contributions presented in this study are included in the article. Further inquiries can be directed to the corresponding author.
